# Generation of knockout rabbits using transcription activator-like effector nucleases

**DOI:** 10.1186/2045-9769-3-3

**Published:** 2014-02-05

**Authors:** Yu Wang, Nana Fan, Jun Song, Juan Zhong, Xiaogang Guo, Weihua Tian, Quanjun Zhang, Fenggong Cui, Li Li, Philip N Newsome, Jon Frampton, Miguel A Esteban, Liangxue Lai

**Affiliations:** 9 Key Laboratory of Regenerative Biology of the Chinese Academy of Sciences and Guangdong Provincial Key Laboratory of Stem Cells and Regenerative Medicine, South China Institute for Stem Cell Biology and Regenerative Medicine, Guangzhou Institutes of Biomedicine and Health, Guangzhou, 510530 China; 10 NIHR Liver BRU and Centre for Liver Research, University of Birmingham, Birmingham, UK; 11 Liver and Hepatobiliary Unit, Queen Elizabeth Hospital Birmingham, Birmingham, UK; 12 Institute for Biomedical Research, College of Medical and Dental Sciences, University of Birmingham, Birmingham, B15 2TT UK

**Keywords:** Rabbits, Animal models, Zinc-finger nucleases, Transcription activator-like effector nucleases, TALENs, Genome editing, Knockout

## Abstract

Zinc-finger nucleases and transcription activator-like effector nucleases are novel gene-editing platforms contributing to redefine the boundaries of modern biological research. They are composed of a non-specific cleavage domain and a tailor made DNA-binding module, which enables a broad range of genetic modifications by inducing efficient DNA double-strand breaks at desired loci. Among other remarkable uses, these nucleases have been employed to produce gene knockouts in mid-size and large animals, such as rabbits and pigs, respectively. This approach is cost effective, relatively quick, and can produce invaluable models for human disease studies, biotechnology or agricultural purposes. Here we describe a protocol for the efficient generation of knockout rabbits using transcription activator-like effector nucleases, and a perspective of the field.

**Electronic supplementary material:**

The online version of this article (doi:10.1186/2045-9769-3-3) contains supplementary material, which is available to authorized users.

## Introduction

### Background

Biomedical research is under growing pressure to approach translation to clinical practice. Before this happens, there are safety concerns that cannot always be addressed by traditional procedures and require the development of suitable animal models. This is particularly obvious in the field of regenerative medicine, where it is expected that yet poorly understood stem cells or their derivatives will be transplanted -sometimes in large numbers- into patients [1]. Unquestionably, rodents (mostly mice) and other small organisms have been instrumental for clarifying the molecular pathways involved in human physiology and disease. Mice are easily handled, relatively inexpensive, and can be used in substantial numbers for providing rigorous statistical analysis. However, their physiology is in general rather different from humans, and their short life span prevents longitudinal studies of safety and efficacy. Accordingly, they frequently fall short of utility for translational research [1]. For example, mouse metabolic and inflammatory responses have poor correlation with the human conditions [2], the mouse and human retina are anatomically and histologically different [1], and knocking out the gene responsible for cystic fibrosis in humans (*CFTR*) reproduces the disease in pigs but not in mice [3]. Thus, there is an urgent need to develop larger and more complex animal models that can bypass these limitations.

### Rabbits in biomedical research

The choice of mid-size/large animal model (e.g., rabbits, cats, dogs, cattle, horses, goats, pigs, or nonhuman primates) for scientific research depends on the disease/condition to be studied, but is also influenced by other considerations including the more or less privileged position in the human community. Rabbits are small herbivore mammals belonging to the order Lagomorpha that are found ubiquitously. They are closer phylogenetically to humans than rodents, measure up to 50 cm in length, and weight 2–5 kg, which makes them big enough for certain procedures but relatively easy to handle. They have as well a long life span (9–12 years), require low cost maintenance, and have short pregnancy period with large offspring. Nowadays, rabbits are extensively used as live bioreactors (for producing polyclonal antibodies or milk enriched in human proteins) [4], for orthopedic [5] and ophthalmic (due to their large eyes with retina composition similar to humans) research [6], and also cardiovascular/metabolic disease studies [7]. The latter is of relevance because cardiovascular and metabolic diseases consume a huge part of the national health cost in any country. In this regard, there are big differences in lipid metabolism between humans and mice, while rabbits are similar to humans in many aspects. For instance, as opposed to humans, mice are highly resistant to diet-induced atherosclerosis due to high levels of high-density lipoproteins (HDL) in plasma, but rabbits are not [7]. Nevertheless, in spite of these and other advantages over other animals, rabbit experimentation beyond the mentioned topics has been limited by the lack of comprehensive tools for genetic engineering. Transgenic rabbits can be generated by microinjection of the desired DNA construct (normally a lentivirus or bacterial artificial chromosome) into a fertilized egg. This has allowed the production of exceptional models such as the mutant rhodopsin and mutant *KCNQ1*/*KCNH2* rabbits, which are employed to study retinitis pigmentosa [6] and long QT syndrome [8], respectively. However, transgene over-expression cannot reproduce many human diseases, and the generation of gene substitutions/knockouts with bacterial artificial chromosomes is rather inefficient.

### Traditional genetic engineering of animals

In the past, the remarkable success of genetic engineering in mice has mostly depended on the isolation and manipulation of embryonic stem cell lines (ESCs) using standard homologous recombination techniques. ESCs can be cultured for prolonged periods of time (thus allowing lengthy handling) in an undifferentiated state, and later on be employed for generating chimeric mice with germline transmission by injection into heterologous blastocysts. Since the isolation of the first mouse ESC line in 1981 [9], there have been many attempts to generate pluripotent stem cell lines from other species. Interestingly, several groups have reported the isolation of rabbit ESC-like cells [10]. These cell lines expressed stem cell-associated markers and maintained apparent pluripotency during multiple passages *in vitro*, but none of them have been convincingly proven to produce chimera. Among the few reported cases, Schoonjans *et al.* described low rate of chimerism (5%) with high contribution based on the coat color [11], and Zakhartchenko *et al.* a single live-born chimera (with low level of mixed coat color) that died shortly [12]. Other groups have produced rabbit induced pluripotent stem cells [13, 14] by reprogramming somatic cells with a cocktail of exogenous transcription factors [15], but these ESC-like cells failed as well to contribute to chimeras or the procedure was not tested. The low rate of chimera formation may be caused by a problem of the ESCs to integrate into the inner cell mass, and/or from failure of the incorporated ESCs to truly participate in embryo development. This deficiency is not a technical issue (e.g., because of the injection procedure or similar), as in fact Gardner and Munro reported the generation of chimeric rabbits by blastocyst injection of heterologous inner cell mass cells as early as in 1974 [16]. Notably, a valuable alternative for genetic modification that is frequently employed in those species for which bona fide ESCs have not been established (e.g. pigs and cattle) is the generation of modified animals by nuclear transfer [17]. This method allows the genetic manipulation of the donor somatic cells prior to transfer into an enucleated oocyte. Yet, it has high frequency of developmental abnormalities and also the caveat that somatic cells have limited lifespan, thus permitting only simple substitutions (e.g., heterozygous knockouts) by means of standard homologous recombination techniques. Moreover, although rabbit nuclear transfer pioneered the field, it is more challenging than for other species and few successful cases have been reported [10].

### Genetic engineering with designer nucleases

**Figure 1:**
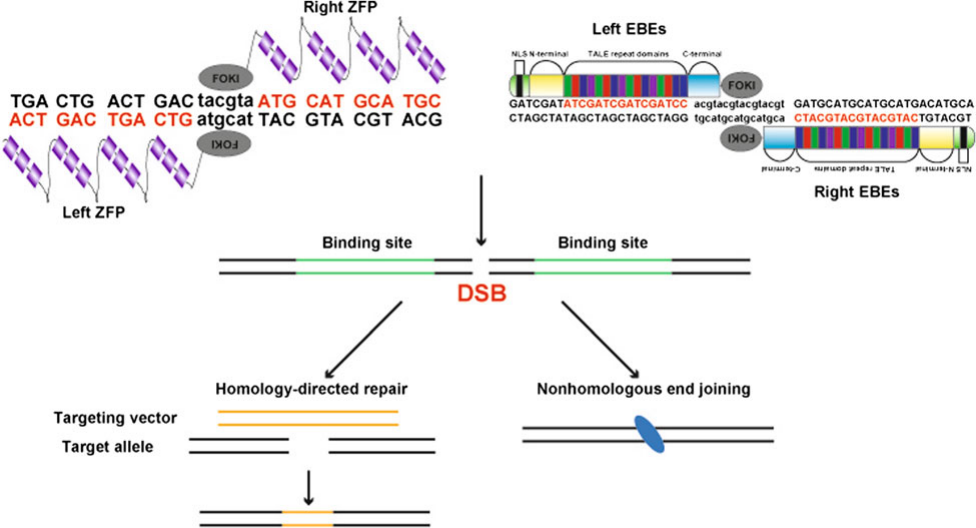
**Schematic depiction of how ZFNs and TALENs recognize target DNA and induce genome editing.** ZFP stands for zinc-finger protein, NLS for nuclear localization signal, N-terminal and C-terminal for amino- and carboxyl-terminal, respectively. DSBs induced by the designer nucleases can be repaired by homology-directed repair or nonhomologous end joining, which can result in knock-ins or knockouts, respectively. FOKI can be substituted by other restriction endonucleases [22].

To overcome the above-mentioned issues, a new technology termed “genome editing” has emerged that allows investigators to modify virtually any gene in a variety of organisms and cell types [18]. Two remarkable examples of this novel approach are zinc-finger nucleases (ZFNs) and transcription activator-like effector (TALE) nucleases (TALENs). These 2 types of designer nucleases are composed of a programmable module that can be adapted to recognize specific genomic sequences, and a non-specific DNA cleavage domain (Figure 1). This combination can produce DNA double-strand breaks (DSBs) at specific loci, which by means of error-prone nonhomologous end joining or homology-directed repair can result in knockouts, nucleotide substitutions, knock-ins, and even larger chromosomal rearrangements [18]. The zinc-finger domain is one of the most frequent motifs in mammalian DNA-binding proteins. Its modular (ββα) structure exposes several amino acids that recognize 3 base pairs in the major groove of DNA [19]. Such unique mode of action made attractive the design of multimodular custom-made DNA-binding proteins with site-specific affinities, which were then fused to the restriction endonuclease FokI [19] and pioneered the field. On the other hand, TALE proteins are naturally occurring proteins from the plant pathogen Xanthomonas (a type of proteobacteria) that contain individual repeats (each typically consisting of 34 amino acids) targeting each a single DNA base pair [20]. Like with ZFNs, TALE repeats can be assembled into a multimodular protein that recognizes contiguous DNA sequences (Figure 1). Yet, the single base recognition by TALE repeats makes the design of TALENs more flexible than ZFNs. In fact, a series of systematized strategies have been developed that enable relatively quick and affordable design/assembly compared to the more tedious and costly ZFNs [18].

To solve the restrictions surrounding traditional genetic engineering of rabbits, we and others have injected mRNA encoding designer nucleases into fertilized rabbit oocytes [21, 22]. Flisikowska *et al.* reported the generation of knockout rabbits for the immunoglobulin M locus using ZFNs [21], and Song *et al.* knockouts for both *Rag 1* and *2* using TALENs [22]. The former model can be used for producing therapeutic human polyclonal antibodies, and the latter for bone marrow gene therapy or cell transplantation studies. Below we describe a step-by-step protocol for producing knockout rabbits using TALENs.

## Basic protocol

### Essential reagents/materials, reagent setup and equipment

Golden Gate TALEN and TAL Effector Kit 2.0 (Addgene, cat. no. 1000000024)

mMESSAGE mMACHINE^®^ T7 Kit (Ambion, cat. no. AM1344)

RNeasy Mini Elute Cleanup Kit (QIAgen, cat. no. 74204)

Tergitol^**®**^ Type NP-40 (Sigma, cat. no. NP40)

10× Taq Buffer (Takara, cat. no. R001A)

Proteinase K (Sigma, cat. no. P2308)

T7 Endonuclease I (New England Biolabs, cat no. M0302S)

Pregnant mare’s serum gonadotropin (Ningbo Renjian Pharmaceutical Co., Ltd., cat. no. 110254564)

Human chorionic gonadotropin (hCG; Ningbo Renjian Pharmaceutical Co., Ltd., cat. no. 110251282)

New Zealand white rabbits (Experimental Animal Center of Southern Medical University, Guangzhou, China)

Medium 199, Hank’s (Gibco, cat. no. 12350)

Fetal bovine serum (Hyclone, cat. no. SH30070.03)

Mineral oil (for embryos) (Thermo Fisher, cat. no. 8042-47-5)

Earle’s balanced salt solution (EBSS; Hyclone, cat. no. SH30029.09)

Essential amino acid solution (Sigma, cat. no. B6766)

Non-essential amino acid solution (Sigma, cat. no. M7145)

L-glutamine (Sigma, cat. no. G8540)

Sodium pyruvate (Sigma, cat. no. P4562)

*Embryo lysis buffer*: mix 1% NP40 and 50 ng/μl proteinase K in 1× Taq buffer.

100× L–glutamine stock solution: Dissolve 0.146 g of L-glutamine in 10 ml of EBSS to make the stock solution. Filter-sterilize with a 0.22-μm syringe filter, then make 200-μl aliquots and store at -20°C for up to 6 months.

100× sodium pyruvate stock solution: Dissolve 0.044 g of sodium pyruvate in 10 ml of EBSS to make the stock solution. Filter-sterilize with a 0.22-μm syringe filter, then make 200-μl aliquots and store at 4°C for up to 1 month.

*Embryo culture medium:* The basic medium contains EBSS supplemented with 1% nonessential amino acids and 2% essential amino acids. It should be stored at 4°C and used for up to 3 weeks. 100 μl of the glutamine stock, 100 μl of the sodium pyruvate stock and 1 ml of fetal bovine serum should be added to 8.8 ml of basic medium before use.

*Embryo manipulation medium:* Medium 199, Hank’s supplemented with 10% fetal bovine serum. Store at 4°C for up to 3 weeks.

Borosilicate glass capillaries (WPI, cat. no. TW100-4; for preparing the holding pipette)

Borosilicate glasses with filament (Sutter, cat. no. BF100-78-10; for preparing the injection pipette)

Micromanipulator set system (Narishige, cat. no. ON3/MP3.3/IPE5.1)

Pipette puller (Sutter, cat. no. P-97)

Microforge (Narishige, cat. no. MF900)

Stereoscopic microscope (Nikon, cat. no. SM2645)

Inverted microscope (Olympus, cat. no. IX71)

Microloader (Eppendorf, cat. no. A246525)

### Procedure

I. TALEN design and preparation. TIME: ~7 days.

**Figure 2:**
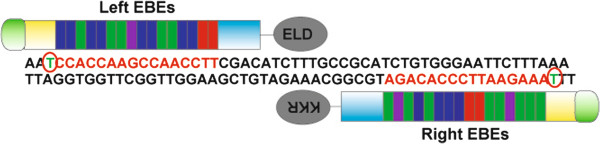
**A Pair of TALENs used for targeting the** ***rabbit Rag 1 gene***. Red circles show a 5′-T preceding the TALEN EBE binding sequences (in red). The spacer has a length of 16 base pairs. We used the FOKI variants ELD and KKR [22].

**Figure 3:**
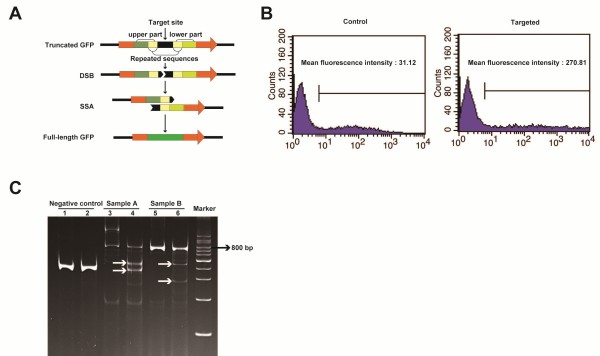
**In vitro and in vivo testing of TALENs. A**. Schematic depiction of the SSA test as explained in step 4 of the procedure. **B**. Flow cytometry analysis of HEK 293 T cells transfected with a GFP “dead” reporter shows increased GFP activity with co-transfection of a specific pair of TALEN plasmids compared to the control. **C**. T7 endonuclease I test result. Lanes 1 and 2 are negative controls, 3 and 5 are samples A and B before treatment, respectively, 4 and 6 are samples A and B after treatment, respectively. White arrows indicate the expected fragments after cleavage by T7 endonuclease I; bp stands for base pairs.

  1. Choose the target gene and locate the genomic DNA sequence using Ensembl (http://www.ensembl.org). For a complete knockout, the target region should normally be within the first few exons (exons 1 or 2) of your gene of interest. The possibility of an additional transcription initiation site or an internal translation initiation site should be considered. We also recommend performing PCR amplification and sequencing of the selected region, as this can prevent mistakes due to incorrect annotation of the rabbit genome or rabbit breed variations. Partial rabbit genome sequences are also included in NCBI (http://www.ncbi.nlm.nih.org). If the target gene were not included in any of the databases then a different strategy (likely involving cloning) will be necessary.
  2. Design the TALEN effector binding elements (EBEs) using standard principles applied in your laboratory. We routinely design 2 pairs of EBEs (15-17 base pairs each) for each target gene. We use the web-based public program TAL Effector Nucleotide Targeter 2.0 (https://tale-nt.cac.cornell.edu) for designing the EBEs. After obtaining candidates with this program, we adhere to 3 main principles derived from the reports by Cermak *et al.* and Doyle *et al.*[20, 23] (Figure 2). First, the EBEs (on the sense and antisense genomic DNA strand) should be preceded by a 5′-T. Second, the average G nucleotide composition of the left EBE should be less than 25%, and the same applies to the average C composition of the right EBE. Third, the optimum spacer length between the 2 EBEs should be 15-17 base pairs.
  3. Assemble the TALENs using the Golden Gate TALEN assembly kit [20, 23]; the details of this method won’t be discussed here. There are other methods available for TALEN assembly including PCR-based modular assembly [24], FLASH assembly [25], as well as commercial approaches.
II. *In vitro* and *in vivo* testing of TALENs.
  4) Single-strand annealing (SSA) detection. The SSA pathway of homologous recombination repairs DSBs between 2 repeated sequences and is used to test TALEN cutting efficiency *in vitro*[26]. TIME: ~5-7 days (not including vector preparation).
    - Construct a “dead” reporter plasmid by inserting the DNA sequence targeted by the TALENs’ EBEs and the spacer sequence (~48 base pairs) into the GFP (green fluorescent protein) coding sequence (Figure 3A). A similar readout can be obtained by creating a “dead” luciferase reporter plasmid [26].
    - Transfect the reporter plasmid together with TALEN plasmids into HEK 293 T cells. If the TALENs produce DSBs then the TALEN target sequence would be removed away by the SSA. This will cause that the cells express GFP, which can be observed with a fluorescence microscope and/or quantified by flow cytometry. The latter can help discern which TALENs display higher activity (Figure 3B). Notably, the results from the GFP or luciferase test don’t represent the real activity of TALENs in living cells or embryos. In fact, epigenetic modifications inducing a closed chromatin conformation (e.g., DNA methylation) are known to reduce TALEN cutting efficiency [27].
  5) T7 endonuclease I test. This enzyme recognizes and cleaves non-perfectly matched DNA. It can thus be employed to confirm the TALENs’ activity *in vivo* because the DSBs produced by TALENs will generate various kinds of indels in the DNA of these embryos. The latter will result in non-perfectly matched DNA heteroduplexes after denaturing and reannealing the PCR products for the region of interest *in vitro*. TIME: ~10-12 hours (not including mRNA preparation and embryo injection).

    - Prepare *in vitro* translated TALEN-coding mRNAs (see step 6 below). Microinject the mRNAs into rabbit embryos (see step 10 below) and harvest the embryos with *embryo lysis buffer* 5 days later.
    - Use this lysate to amplify the DNA sequence containing the TALEN target region by PCR.
    - Subject the PCR product to denaturation and reannealing to obtain DNA heteroduplexes. Incubate the product with T7 endonuclease I at 37°C for 15 minutes, and stop the reaction with 0.5 M EDTA. The mixture is then ready for electrophoresis on a polyacrylamide gel. If the TALENs are effective there should be at least 3 bands (the original PCR product and 2 bands resulting from the T7 endonuclease I cleavage), rather than just 1 (Figure 3C).
III. *In vitro* transcription of TALEN-coding mRNAs. TIME: ~1 day.
  6) Choose one pair of TALENs with high activity. Linearize the corresponding plasmids with an appropriate restriction enzyme and transcribe into mRNA using the mMESSAGE mMACHINE^®^ T7 Kit from Ambion. Please note that the backbone vector expressing the TALENs should contain the appropriate promoter and polyA signal for *in vitro* transcription. Purify the mRNAs using RNeasy Mini Elute Cleanup Kit. Run on an electrophoresis gel to check the size and integrity of the mRNAs, and calculate the concentration with a spectrophotometer. All materials should be RNase free and the procedure performed cautiously to avoid RNA degradation. The samples can be aliquoted at a concentration of 50 ng/μl, stored at -80°C, and thawed for use when the embryos are ready. Only 1 process of freezing/thawing is recommended.
IV. Microinjection of embryos with TALEN-coding mRNAs.
  7) Preparation of micromanipulation pipettes [28]. TIME: ~15-30 minutes.

**Figure 4:**
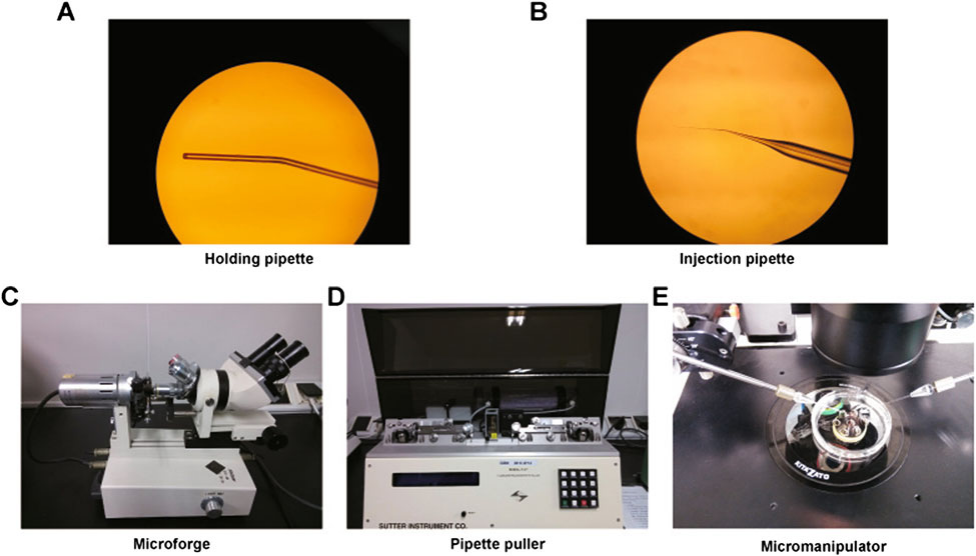
**Photographs of holding and injection pipettes, microforge, pipette puller and micromanipulator. A**. Holding pipette. **B**. Injection pipette. **C**. Microforge. **D**. Pipette puller. **E**. Micromanipulator. The holding and injection pipettes can be observed on both sides of the micromanipulator.

    - Holding pipette (Figure 4A). Pull out the borosilicate glass capillaries on the flame of an alcohol lamp, then break at the tip leaving an outside diameter of ~120-180 μm. Blunt the broken end until the inner diameter is reduced to ~20-30 μm. Bend the pipette close to the blunt end (about 300 μm back) at 30 degrees with a microforge (Figure 4C).
    - Injection pipette (Figure 4B). Pull out the borosilicate glass using a pipette puller (Figure 4D) and the following parameters: pressure 200, heat 505, pull 95, velocity 70, and time-delay 80. Bend the pipette close to the tip (about 400 μm back) at 30 degrees with a microforge. The injection pipette should be prepared on the same day or the day before embryo injection, but not beforehand because of tendency to become obstructed.
  8) Preparation of rabbit embryos. TIME: ~6 days.
    - Inject at least 2 donor female rabbits intramuscularly with 100 IU of pregnant mare’s serum gonadotropin. Mate them after 72-120 hours, and then inject 100 IU of hCG intravenously. Simultaneously, inject additional female rabbits (we routinely prepare 2-3 additional ones for each pair of TALENs) with 100 IU of hCG. These extra rabbits will be used as surrogate mothers (see step 11 below).
    - Prepare a 35 mm tissue culture dish containing multiple droplets of *embryo culture medium*, and a 4-well plate with each well containing 500 μl of *embryo culture medium*. Cover them with mineral oil and place them in a 5% CO_2_ incubator at 38.5°C (body temperature in rabbits) to balance for at least 3 hours before use.
    - Sacrifice the donor rabbits 18-20 hours after hCG injection. Flush the fertilized oocytes from the oviducts with pre-warmed (at 38.5°C) *embryo manipulation medium*. Pick pronuclear-stage embryos and wash them for 3 times in the droplets of *embryo culture medium* prepared beforehand (see step 8b above). Transfer the washed embryos to the 4-well plate containing *embryo culture medium* (see step 8b above) and then put it back to the incubator before use.
  9) Micromanipulator set-up. TIME: ~10 minutes.
    - Mix (at 1:1 ratio) the paired TALEN-coding mRNA solutions (each at a concentration of 50 ng/μl). Load the mixed mRNA solution into the open end of the injection pipette with a microloader. Attach the injection pipette to one of the holders of the micromanipulator (Figure 4E).
    - Attach the holding pipette to the other holder of the micromanipulator (Figure 4E).
    - Prepare several droplets of pre-warmed *embryo manipulation medium* in a 60 mm tissue culture dish and cover them with mineral oil (Figure 4E). Adjust the holding and injection pipettes to the center of a droplet (Figure 4E). Gently break the tip of the injection pipette by hitting the holding pipette (Additional file 1).
  10) Embryo microinjection. TIME: ~10 minutes.
    - Take the 4-well plate containing embryos in *embryo culture medium* (see step 8c above). Put 30-40 pronuclear-stage embryos into a single manipulation droplet in the 60 mm dish (see step 9c above).
    - Adjust the holding and injection pipettes to pick and inject the embryos individually with 5-10 pl of the TALEN-coding mRNA solution. It is important to adjust the flow rate of the mRNA solution beforehand by injecting first into the perivitelline space (the space between the zona pellucida and the cell membrane) of one embryo; then inject sequentially into the cytoplasm of each embryo. The latter should produce slight swelling (Additional file 2).
    - Wash the injected embryos 3 times in the droplets of *embryo culture medium* prepared beforehand (see step 8b above) and transfer to the 4-well plate with *embryo culture medium* (see step 8b above). Place the 4-well plate back in the incubator.
    - Half of the injected embryos will be lysed and employed for confirming that the selected TALENs cut the target sequence *in vivo* (see step 5 above). These embryos need to be maintained in the same 4-well plate with *embryo culture medium* (see step 10b above) in the incubator until they reach the blastocyst stage (normally 4-5 days). The other half will be transferred into the foster mother after letting them recover for 2-3 hours in the same 4-well plate with *embryo culture medium* inside the incubator. We perform both procedures (*in vivo* testing of TALEN cutting efficiency and transfer to foster mother) simultaneously in our laboratories, but if the methodology has just been set up then it may be advisable to optimize (e.g., modifying the mRNA concentration) the cutting efficiency first.
V. Embryo transfer to foster mother.
  11) Transfer 8-14 good quality (as observed under the microscope) embryos through the infundibulum of the oviduct of each surrogate mother. TIME: 2-4 hours.
  12) Detect pregnancy (by palpation) around 15 days after embryo transfer. The rabbits will be born by spontaneous delivery at around day 30 of pregnancy.
VI. Knockout detection of newborn rabbits. TIME: 5-7 days.
  13) Extract genomic DNA of newborn rabbits using ear tissue and the appropriate lysis buffer. Amplify the region of interest by PCR; a nested PCR reaction can be employed if the desired band is difficult to amplify. Use T-A cloning and then sequencing to confirm the nature of the mutation. Of note, if the TALENs acted during or after the 2-cell stage the rabbits may be chimeric, in which case the offspring will be knockouts only if there is germline transmission.
VII. Off-target analysis. TIME: 1-2 weeks.
  14) To determine the TALEN specificity, we use the online e-PCR program (hhttp://www.ncbi.nlm.nih.gov/projects/e-pcr/) in NCBI. We routinely choose potential off-target sites with no more than 100 base pairs between the left and right TALEN EBEs. In our previous study, we designed primers for 2 and 6 potential off-target sites of *Rag1* and *Rag2* TALENs, respectively. The corresponding PCR products were sequenced and compared with the original sequence, and showed no evidence of off-target effects [22].

## Conclusions and future perspectives

The protocol described here allows the generation of knockout rabbits using TALENs in ~6-8 weeks. This methodology will expand the current uses of rabbits for biomedical purposes, as they have significant advantages compared with both rodents and larger animals including pigs or cattle. Specifically, we anticipate a bright future for immunodeficient rabbits in stem cell transplantation, and as a preclinical model for gene therapy in humans. A limitation of rabbits, and other non-rodent species as well, is the lack of comprehensive materials for their study (e.g., antibodies and gene expression arrays) [1] and their incompletely sequenced and/or poorly annotated genome. On the other hand, rabbits and other larger animals will likely be instrumental to develop new techniques (e.g., surgical and imaging) and methodologies (e.g., protocols for expanding/injecting cells or gene-therapy viral vectors) similar to those that could be eventually required in humans. For more complex genetic modifications (e.g., gene knock-in) than the 2 studies reported thus far using ZFNs and TALENs [21, 22], it is possible to simultaneously inoculate rabbit fertilized eggs with designer nucleases and donor plasmids/single-stranded oligonucleotides [29]. In addition, there is a new type of genome editing platform not yet reportedly tested in rabbits, the clustered regularly interspaced short palindromic repeats (CRISPRs) and their associated (Cas) proteins, which allows the simultaneous modification of multiple genes [30]. CRISPRs/Cas are based on an antiviral defense system in bacteria, and their design/preparation is even simpler and less time consuming than for TALENs. Moreover, it has being reported that in at least some instances CRISPRs/Cas can successfully target genes for which TALENs display low cutting efficiency [31]. At this point, there is no clear consensus regarding the risk of off-target effects with each of the genome-editing platforms (ZFNs, TALENs and CRISPRs/Cas). Nevertheless, these technologies are evolving [32] and this issue will likely be significantly minimized in the near future. Besides all these technical considerations, it will be important to revise regulatory and ethical issues, as research worldwide on these modified mid-size and large animal models should be compliant with the highest standards of animal care and husbandry.

## Electronic supplementary material

Additional file 1: **Preparation of the injection pipette (video).** (AVI 652 KB)

Additional file 2: **Embryo injection (video).** (AVI 3 MB)

## References

[1] Harding J, Roberts RM, Mirochnitchenko O (2013). Large animal models for stem cell therapy. Stem Cell Res Ther.

[2] Seok J, Warren HS, Cuenca AG, Mindrinos MN, Baker HV, Xu W, Richards DR, McDonald-Smith GP, Gao H, Hennessy L, Finnerty CC, Lopez CM, Honari S, Moore EE, Minei JP, Cuschieri J, Bankey PE, Johnson JL, Sperry J, Nathens AB, Billiar TR, West MA, Jeschke MG, Klein MB, Gamelli RL, Gibran NS, Brownstein BH, Miller-Graziano C, Calvano SE, Mason PH (2013). Genomic responses in mouse models poorly mimic human inflammatory diseases. Proc Natl Acad Sci U S A.

[3] Rogers CS, Stoltz DA, Meyerholz DK, Ostedgaard LS, Rokhlina T, Taft PJ, Rogan MP, Pezzulo AA, Karp PH, Itani OA, Kabel AC, Wohlford-Lenane CL, Davis GJ, Hanfland RA, Smith TL, Samuel M, Wax D, Murphy CN, Rieke A, Whitworth K, Uc A, Starner TD, Brogden KA, Shilyansky J, McCray PB, Zabner J, Prather RS, Welsh MJ (2008). Disruption of the CFTR gene produces a model of cystic fibrosis in newborn pigs. Science.

[4] Chrenek P, Ryban L, Vetr H, Makarevich AV, Uhrin P, Paleyanda RK, Binder BR (2007). Expression of recombinant human factor VIII in milk of several generations of transgenic rabbits. Transgenic Res.

[5] Hu J, Qu J, Xu D, Zhang T, Qin L, Lu H (2013). Combined application of low-intensity pulsed ultrasound and functional electrical stimulation accelerates bone-tendon junction healing in a rabbit model. J Orthop Res.

[6] Kondo M, Sakai T, Komeima K, Kurimoto Y, Ueno S, Nishizawa Y, Usukura J, Fujikado T, Tano Y, Terasaki H (2009). Generation of a transgenic rabbit model of retinal degeneration. Invest Ophthalmol Vis Sci.

[7] Wang Y, Niimi M, Nishijima K, Waqar AB, Yu Y, Koike T, Kitajima S, Liu E, Inoue T, Kohashi M, Keyamura Y, Yoshikawa T, Zhang J, Ma L, Zha X, Watanabe T, Asada Y, Chen YE, Fan J (2013). Human apolipoprotein A-II protects against diet-induced atherosclerosis in transgenic rabbits. Arterioscler Thromb Vasc Biol.

[8] Marian AJ, Wu Y, Lim DS, McCluggage M, Youker K, Yu QT, Brugada R, DeMayo F, Quinones M, Roberts R (1999). A transgenic rabbit model for human hypertrophic cardiomyopathy. J Clin Invest.

[9] Evans MJ, Kaufman MH (1981). Establishment in culture of pluripotential cells from mouse embryos. Nature.

[10] Tancos Z, Nemes C, Polgar Z, Gocza E, Daniel N, Stout TA, Maraghechi P, Pirity MK, Osteil P, Tapponnier Y, Markossian S, Godet M, Afanassieff M, Bosze Z, Duranthon V, Savatier P, Dinnyes A (2012). Generation of rabbit pluripotent stem cell lines. Theriogenology.

[11] Schoonjans L, Albright GM, Li JL, Collen D, Moreadith RW (1996). Pluripotential rabbit embryonic stem (ES) cells are capable of forming overt coat color chimeras following injection into blastocysts. Mol Reprod Dev.

[12] Zakhartchenko V, Flisikowska T, Li S, Richter T, Wieland H, Durkovic M, Rottmann O, Kessler B, Gungor T, Brem G, Kind A, Wolf E, Schnieke A (2011). Cell-mediated transgenesis in rabbits: chimeric and nuclear transfer animals. Biol Reprod.

[13] Honda A, Hirose M, Hatori M, Matoba S, Miyoshi H, Inoue K, Ogura A (2010). Generation of induced pluripotent stem cells in rabbits: potential experimental models for human regenerative medicine. J Biol Chem.

[14] Osteil P, Tapponnier Y, Markossian S, Godet M, Schmaltz-Panneau B, Jouneau L, Cabau C, Joly T, Blachere T, Gocza E, Bernat A, Yerle M, Acloque H, Hidot S, Bosze Z, Duranthon V, Savatier P, Afanassieff M (2013). Induced pluripotent stem cells derived from rabbits exhibit some characteristics of naive pluripotency. Biol Open.

[15] Cai J, Zhang Y, Liu P, Chen S, Wu X, Sun Y, Li A, Huang K, Luo R, Wang L (2013). Generation of tooth-like structures from integration-free human urine induced pluripotent stem cells. Cell Regeneration.

[16] Gardner RL, Munro AJ (1974). Successful construction of chimaeric rabbit. Nature.

[17] Lai L, Kolber-Simonds D, Park KW, Cheong HT, Greenstein JL, Im GS, Samuel M, Bonk A, Rieke A, Day BN, Murphy CN, Carter DB, Hawley RJ, Prather RS (2002). Production of alpha-1,3-galactosyltransferase knockout pigs by nuclear transfer cloning. Science.

[18] Gaj T, Gersbach CA, Barbas CF (2013). ZFN, TALEN, and CRISPR/Cas-based methods for genome engineering. Trends Biotechnol.

[19] Kim YG, Cha J, Chandrasegaran S (1996). Hybrid restriction enzymes: zinc finger fusions to Fok I cleavage domain. Proc Natl Acad Sci U S A.

[20] Cermak T, Doyle EL, Christian M, Wang L, Zhang Y, Schmidt C, Baller JA, Somia NV, Bogdanove AJ, Voytas DF (2011). Efficient design and assembly of custom TALEN and other TAL effector-based constructs for DNA targeting. Nucleic Acids Res.

[21] Flisikowska T, Thorey IS, Offner S, Ros F, Lifke V, Zeitler B, Rottmann O, Vincent A, Zhang L, Jenkins S, Niersbach H, Kind AJ, Gregory PD, Schnieke AE, Platzer J (2011). Efficient immunoglobulin gene disruption and targeted replacement in rabbit using zinc finger nucleases. PLoS One.

[22] Song J, Zhong J, Guo X, Chen Y, Zou Q, Huang J, Li X, Zhang Q, Jiang Z, Tang C, Yang H, Liu T, Li P, Pei D, Lai L (2013). Generation of RAG 1- and 2-deficient rabbits by embryo microinjection of TALENs. Cell Res.

[23] Doyle EL, Booher NJ, Standage DS, Voytas DF, Brendel VP, Vandyk JK, Bogdanove AJ (2012). TAL Effector-Nucleotide Targeter (TALE-NT) 2.0: tools for TAL effector design and target prediction. Nucleic Acids Res.

[24] Zhang F, Cong L, Lodato S, Kosuri S, Church GM, Arlotta P (2011). Efficient construction of sequence-specific TAL effectors for modulating mammalian transcription. Nat Biotechnol.

[25] Reyon D, Tsai SQ, Khayter C, Foden JA, Sander JD, Joung JK (2012). FLASH assembly of TALENs for high-throughput genome editing. Nat Biotechnol.

[26] Sakuma T, Hosoi S, Woltjen K, Suzuki K, Kashiwagi K, Wada H, Ochiai H, Miyamoto T, Kawai N, Sasakura Y, Matsuura S, Okada Y, Kawahara A, Hayashi S, Yamamoto T (2013). Efficient TALEN construction and evaluation methods for human cell and animal applications. Genes Cells.

[27] Bultmann S, Morbitzer R, Schmidt CS, Thanisch K, Spada F, Elsaesser J, Lahaye T, Leonhardt H (2012). Targeted transcriptional activation of silent oct4 pluripotency gene by combining designer TALEs and inhibition of epigenetic modifiers. Nucleic Acids Res.

[28] Kishigami S, Wakayama S, Thuan NV, Ohta H, Mizutani E, Hikichi T, Bui HT, Balbach S, Ogura A, Boiani M, Wakayama T (2006). Production of cloned mice by somatic cell nuclear transfer. Nat Protoc.

[29] Chen F, Pruett-Miller SM, Huang Y, Gjoka M, Duda K, Taunton J, Collingwood TN, Frodin M, Davis GD (2011). High-frequency genome editing using ssDNA oligonucleotides with zinc-finger nucleases. Nat Methods.

[30] Wei C, Liu J, Yu Z, Zhang B, Gao G, Jiao R (2013). TALEN or Cas9 - rapid, efficient and specific choices for genome modifications. J Genet Genomics.

[31] Ding Q, Regan SN, Xia Y, Oostrom LA, Cowan CA, Musunuru K (2013). Enhanced efficiency of human pluripotent stem cell genome editing through replacing TALENs with CRISPRs. Cell Stem Cell.

[32] Ran FA, Hsu PD, Lin CY, Gootenberg JS, Konermann S, Trevino AE, Scott DA, Inoue A, Matoba S, Zhang Y, Zhang F (2013). Double nicking by RNA-guided CRISPR Cas9 for enhanced genome editing specificity. Cell.

